# Two Neuroanatomical Signatures in Schizophrenia: Expression Strengths Over the First 2 Years of Treatment and Their Relationships to Neurodevelopmental Compromise and Antipsychotic Treatment

**DOI:** 10.1093/schbul/sbad040

**Published:** 2023-04-12

**Authors:** Stefan du Plessis, Ganesh B Chand, Guray Erus, Lebogang Phahladira, Hilmar K Luckhoff, Retha Smit, Laila Asmal, Daniel H Wolf, Christos Davatzikos, Robin Emsley

**Affiliations:** Department of Psychiatry, Faculty of Medicine and Health Sciences, Stellenbosch University, Tygerberg Campus, Cape Town, South Africa; Center for Biomedical Image Computing and Analytics, Perelman School of Medicine, University of Pennsylvania, Philadelphia; Department of Radiology and Mallinckrodt Institute of Radiology, Washington University School of Medicine, St. Louis; Center for Biomedical Image Computing and Analytics, Perelman School of Medicine, University of Pennsylvania, Philadelphia; Department of Psychiatry, Faculty of Medicine and Health Sciences, Stellenbosch University, Tygerberg Campus, Cape Town, South Africa; Department of Psychiatry, Faculty of Medicine and Health Sciences, Stellenbosch University, Tygerberg Campus, Cape Town, South Africa; Department of Psychiatry, Faculty of Medicine and Health Sciences, Stellenbosch University, Tygerberg Campus, Cape Town, South Africa; Department of Psychiatry, Faculty of Medicine and Health Sciences, Stellenbosch University, Tygerberg Campus, Cape Town, South Africa; Center for Biomedical Image Computing and Analytics, Perelman School of Medicine, University of Pennsylvania, Philadelphia; Center for Biomedical Image Computing and Analytics, Perelman School of Medicine, University of Pennsylvania, Philadelphia; Department of Psychiatry, Faculty of Medicine and Health Sciences, Stellenbosch University, Tygerberg Campus, Cape Town, South Africa

**Keywords:** structural MRI, semi-supervised machine learning, first-episode, long-acting injectable antipsychotic

## Abstract

**Background and Hypothesis:**

Two machine learning derived neuroanatomical signatures were recently described. Signature 1 is associated with widespread grey matter volume reductions and signature 2 with larger basal ganglia and internal capsule volumes. We hypothesized that they represent the neurodevelopmental and treatment-responsive components of schizophrenia respectively.

**Study Design:**

We assessed the expression strength trajectories of these signatures and evaluated their relationships with indicators of neurodevelopmental compromise and with antipsychotic treatment effects in 83 previously minimally treated individuals with a first episode of a schizophrenia spectrum disorder who received standardized treatment and underwent comprehensive clinical, cognitive and neuroimaging assessments over 24 months. Ninety-six matched healthy case–controls were included.

**Study Results:**

Linear mixed effect repeated measures models indicated that the patients had stronger expression of signature 1 than controls that remained stable over time and was not related to treatment. Stronger signature 1 expression showed trend associations with lower educational attainment, poorer sensory integration, and worse cognitive performance for working memory, verbal learning and reasoning and problem solving. The most striking finding was that signature 2 expression was similar for patients and controls at baseline but increased significantly with treatment in the patients. Greater increase in signature 2 expression was associated with larger reductions in PANSS total score and increases in BMI and not associated with neurodevelopmental indices.

**Conclusions:**

These findings provide supporting evidence for two distinct neuroanatomical signatures representing the neurodevelopmental and treatment-responsive components of schizophrenia.

## Introduction

The neurodevelopmental and dopamine (DA) hypotheses are two major theories of schizophrenia that propose to explain the origins and underlying neurobiology of the disorder respectively.^1^ These hypotheses are broadly consistent with the proposal over 40 years ago by Crow^2^ of two distinct syndromes for schizophrenia, each with a specific pathological process. Crow proposed that “acute schizophrenia” is characterized by positive symptoms and is responsive to antipsychotic medication, while the “defect state” is characterized by negative symptoms, intellectual impairment, a poor long-term outcome and structural brain changes. However, while brain morphological differences have been consistently reported in schizophrenia,^3^ attempts to link these changes to both neurodevelopmental compromise^4^ and psychotic symptoms associated with DA dysregulation^5^ have met with limited success. This is not surprising given the heterogeneous nature of the illness with large variation in many aspects including brain structural changes.^6^ Additionally, differences in study populations and methodology including scanning protocols and selection of brain regions, likely contribute to the lack of consistent findings. A better understanding of these associations may further elucidate the underlying neurobiology of the illness and provide clinicians with tools for predicting outcome and individualizing treatment.

In a recent development using novel machine learning methods on regional brain volumetric measures to eliminate confounding variations, two markedly distinct and reproducible neuroanatomical signatures of schizophrenia were identified.^7,8^ Signature 1 is characterized by widespread lower grey matter volumes, particularly in the thalamus, nucleus accumbens, medial temporal, medial prefrontal/frontal and insular cortices, while signature 2 had larger basal ganglia and internal capsule but normal cortical anatomy. It was proposed that signature 1 is related to the neurodevelopmental component of the illness and signature 2 is related to functional abnormalities, perhaps in DA systems, leading secondarily to basal ganglia enlargement. These studies were cross-sectional and conducted in patients in different stages of illness and with varying medication exposure.

In this study, we applied these neuroanatomical signatures to a unique patient cohort to investigate their relationships with both indicators of neurodevelopmental deviance and with clinical and treatment effects. Our aims were 2-fold: to investigate the relationships of the two signatures with indicators of neurodevelopmental compromise and to assess their associations with treatment effects. For this study we examined the signatures as continuous variables rather than categorizing participants into subtypes as firstly, the signatures are not mutually exclusive but rather co-exist in individuals with different expression strengths and secondly, we were interested in assessing dynamic changes in the signature strengths over time. We measured the signature expression strengths in patients with a first episode of schizophrenia with no or minimal prior antipsychotic exposure, assessed their temporal stability over the first two years of treatment and investigated their relationships to selected factors associated with neurodevelopmental compromise (ie, factors implicated in disrupting normal neurodevelopment) and to antipsychotic treatment. Patients received treatment following a specified protocol with a long-acting injectable antipsychotic and underwent comprehensive clinical, cognitive, and neuroimaging assessments at fixed timepoints. This allowed us to investigate pre-treatment effects, to quantify the antipsychotic dose with precision and to assess longitudinal changes in signature expression in relation to treatment. In a recent study using similar methodology in an overlapping sample we found increased basal ganglia volumes that were associated with greater symptom reductions, weight gain, and more extrapyramidal symptoms. Additionally, although not differing significantly from controls, white matter volume increases were associated with greater clinical improvements, weight gain, and extrapyramidal symptoms, and slight cortical thickness reductions were unrelated to treatment.^9^ Based on these findings we hypothesized that signature 1 expression would be related to factors associated with neurodevelopmental compromise, would be stable over two years and would be uninfluenced by antipsychotic treatment. We further hypothesized that signature 2 expression would be unrelated to neurodevelopmental compromise but would be influenced by antipsychotic treatment in terms of dose, efficacy, and emergent side effects.

## Methods

### Study Sample

This single-site, case–control, prospective study was conducted between 2007 and 2017 and assessed clinical, cognitive, and brain-imaging changes over the first 2 years of treatment in patients with a first episode of a schizophrenia spectrum disorder. Previously published results from this cohort are provided in Supplementary Appendix 1. Of note is a recent publication reporting structural brain changes in relation to treatment effects in an overlapping sample to the present one.^9^ Patients were recruited from psychiatric community clinics and first admissions to hospitals within Cape Town and surrounding districts. Participants provided written, informed consent, and we obtained ethics approval from the Human Research Ethics Committee of Stellenbosch University. Eligibility criteria were men and women, in- or out-patients, aged 16–45 years, meeting Diagnostic and Statistical Manual of Mental Disorders, Fourth Edition (DSM-IV) (American Psychiatric Association, 1994) diagnostic criteria for schizophreniform disorder, schizophrenia or schizoaffective disorder. Exclusion criteria were lifetime exposure to > 4 weeks of antipsychotic medication, previous treatment with a long-acting injectable antipsychotic, serious or unstable medical condition or intellectual disability. The case–controls comprised healthy volunteers from the same community, matched by age, gender, and ethnicity. The patients and controls were assessed with the Structured Clinical Interview for DSM-IV (SCID).^10^ Controls were excluded if they had a DSM-IV axis I or II disorder or a first-degree family member with a psychotic disorder.

### Predictors of Neurodevelopmental Compromise

Based on previous reports of associations with disrupted neurodevelopment in schizophrenia, we selected the following variables as putative indicators of neurodevelopmental compromise: A history of schizophrenia in a first-degree relative,^11^ obstetric complications,^12^ childhood trauma^13^ according to the Childhood Trauma Questionnaire (CTQ),^14^ premorbid functioning^15^ according to the Premorbid Adjustment Scale (PAS),^16^ scholastic achievement (highest school grade successfully completed), neurological soft signs^17^ as assessed by the Neurological Evaluation Scale (NES)^18^ and cognitive function^19^ as assessed by the MATRICS (Measurement and Treatment Research to Improve Cognition in Schizophrenia) Cognitive Consensus Battery (MCCB).^20^

### Treatment Related Effects

We considered three aspects of treatment, namely antipsychotic dose, efficacy, and side effects. We calculated the precise antipsychotic dose in flupenthixol mg equivalents at each timepoint using consensus-derived guidelines for dose-equivalencies.^21^ For efficacy we assessed symptom severity with the Positive and Negative Syndrome Scale (PANSS);^22^ for side effects we assessed body mass index (BMI) calculated as kg/m^2^ and for motor side effects we used the Extrapyramidal Symptom Rating Scale (ESRS).^23^ Metabolic assessments comprised fasting blood glucose, high-density lipoprotein (HDL) cholesterol, low-density lipoprotein (LDL) cholesterol, triglycerides and total cholesterol. The clinical, cognitive, laboratory, and MRI measures were assessed at baseline, month 12, and month 24. Urine toxicology tests for cannabis were conducted at months 0, 3, 6, 12, 18, and 24 and the number of positive tests was used as a proxy for frequency of use.

### Treatment

Patients received treatment according to a standard protocol with the lowest possible antipsychotic dose. Oral flupenthixol 1–3 mg/day was prescribed for 1 week and thereafter flupenthixol decanoate intramuscular injections were administered 2-weekly for the study duration. Starting intramuscular dose was 10 mg 2-weekly, with 6-weekly 10 mg increments as required to a maximum of 30 mg 2-weekly. Other antipsychotics, mood stabilizers and psychostimulants were forbidden. Permitted medications included lorazepam, anticholinergics, propranolol, antidepressants and medications for general medical conditions. Five patients were initially treated for 12 weeks with long-acting risperidone injection at a starting dose of 25 mg intramuscular 2-weekly before switching to flupenthixol decanoate. Flupenthixol is pharmacologically similar to several new generation antipsychotics, binding primarily at the D1, D2, D3 receptors, 5-HT2A and 5-HT2C and alpha1-adrenergic receptors and its decanoate formulation has been extensively used in community settings.^24,25^

### Image Acquisition and Pre-processing

Participants were scanned using a T1 ME-MPRAGE weighted structural sequence (TR = 2530 ms; TE_1_ = 1.53 ms TE_2_ = 3.21 ms, TE_3_ = 4.89 ms, TE_4_ = 6.57 ms, flip-angle: 7°C, FoV: 256 mm, 128 slices, 1 mm isotropic voxel size).^26^ Scans were segmented using a multi-atlas region segmentation utilizing an ensemble of registration algorithms and parameters, as described in detail elsewhere (MUSE).^27^ In brief, each individual’s T1 was segmented into 145 anatomical regions of interest from the grey matter, white matter, and cerebrospinal fluid volumes (Supplementary table 1). Volumetric maps were generated voxel-wise^28^ for grey matter and white matter by a deformable registration of the skull stripped T1s in MNI space.^29^

### Identifying Schizophrenia Imaging Signatures and Defining Subgroups

We used the heterogeneity through discriminative analysis (HYDRA) tool to identify the presence and expression strength of the schizophrenia imaging signatures. HYDRA is a semi-supervised machine learning algorithm that uses pre-specified patient and control labels in a data-driven approach to perform classification and clustering *within* the patient group. HYDRA allows for the separation of distinct patient groups rather than forcing patient data into a single common discriminative pattern. The HYDRA parameters were derived from the independent PHENOM consortium dataset (Psychosis Heterogeneity Evaluated via Dimensional Neuroimaging)^7^ and applied to the current dataset to estimate neuroanatomical signature expression strength (E1 and E2). Since controls are assigned a “−1” and schizophrenia patients a “+1” during HYDRA training, a positive E represents the presence of a signature, and a negative E represents its relative absence as described in previous work.^8^ Subgroups were determined accordingly, ie, subgroup 1 “S1” (E1 > 0, E2 < 0), subgroup 2 “S2” (E1 < 0, E2 > 0), both groups “S1+S2” (E1 > 0, E2 > 0) or neither group “S0” (E1 < 0, E2 < 0).

### Statistical Analyses

The analysis set comprised a modified intent-to-treat population, which was participants with clinical data at baseline and at least one suitable MRI scan. Statistical analyses were performed with Statistica version 13.0 (Dell, 2015).

### Primary Analyses

We used linear mixed effect repeated measures for fitting models (MMRM) to compare the visit-wise signature expression strength in patients versus controls. Signature 1 and 2 expression values were entered as the dependent variables in separate models, as repeated measures. We specified intercepts for participants as a random effect. Age and gender were covariates and time was a grouping variable. The group*time interaction was a fixed effect. We then conducted further MMRM analyses in the patients only to assess relationships of the signatures with predictors of neurodevelopmental compromise and treatment effects. Signature expression strength values were the dependent variables and modeled as repeated measures, time was a grouping variable and intercepts for participants were entered as a random effect. We first considered covariates comprising age, gender, ethnicity, duration of untreated psychosis, diagnosis, the number of positive cannabis urine tests, previous antipsychotic exposure (yes/no) and number of days of previous treatment. None had significant effects and were therefore not included in the subsequent models. We then assessed whether our selected predictors of neurodevelopmental compromise had significant effects on signature expression strength. Family history of schizophrenia, history of obstetric complications, CTQ Total score, highest educational achievement and PAS General score were time-invariant predictors, while NES Total score and MCCB composite score were time-dependent predictors. In the final model we investigated relations between treatment effects and signature expression strength. Antipsychotic dose, PANSS Total score, ESRS Total score, and BMI were time-dependent predictors. Fisher’s Least Significant Difference (LSD) test was used post hoc to compare within-group differences for the MMRM analyses, and we used Benjamini and Hochberg false discovery rate (FDR)^30^ to correct for multiplicity with a threshold of < 0.05 for the fixed effect tests. The adjusted significance level was 0.0059. The direction of the effects was established by partial correlational analyses.

### Secondary Analyses

In a set of exploratory analyses, we investigated relationships between signature expression and neurodevelopmental compromise and treatment effects in greater detail. MMRM analyses were conducted in the patients with signature expression strength as the dependent variable and modeled as repeated measures, and intercepts for participants as a random effect. For predictors of neurodevelopmental compromise, we assessed CTQ subscale scores for emotional, physical and sexual abuse and emotional and physical neglect; premorbid adjustment by developmental stage (PAS scores for childhood, early adolescence, late adolescence); NES subscales of sensory integration, motor coordination and motor sequencing; and MCCB cognitive domains of speed of processing, attention/vigilance, working memory, verbal learning, visual learning, reasoning and problem solving and social cognition. For treatment effects, we assessed symptom changes in PANSS positive, negative and disorganized domains as determined by factor analysis,^31^ and fasting blood glucose, HDL-cholesterol, LDL-cholesterol, triglycerides, and total cholesterol. The secondary analyses were designated as exploratory, and corrections for multiplicity were not applied. These findings should therefore be regarded preliminary and suggestive of hypotheses for further studies.

### Sensitivity Analysis

To test whether our results were not an artifact of the modified intent-to-treat population we repeated the primary analyses on the completer population only, ie, those participants who completed the 2 years of follow up and had a month 24 scan.

## Results

Of 126 patients entered, 83 had baseline clinical data and at least one suitable scan. Forty-eight (58%) were antipsychotic naïve at baseline and 35 (42%) had received antipsychotics for a mean ± SD 9.7 ± 6.5 days before study entry. Forty-four patients completed the study. They did not differ significantly from the rest of the sample in terms of age (25.5 ± 7.4 vs 23.8 ± 6.0 years, *P* = .2503), gender (77% vs 71% male, *P* = .4915), highest school grade attained (9.8 ± 2.2 vs 9.9 ± 2.1, *P* = .7411), baseline PANSS total score (93.3 ± 14.2 vs 92.7 ± 15.7, *P* = .8626) and baseline expression strength for signature 1 (0.0015 ± 1.1330 vs −1.2396 ± 1303, *P* = .4095) and signature 2 (−0.7042 ± 1.1074 vs −0.8937 ± 1.1560, *P* = .4479). The control-group comprised 96 healthy volunteers. For patients and controls respectively, the mean age was 24.7 ± 6.8 and 26.0 ± 7.4 years (*P* = .1969), the percentage males was 74% and 62% (*P* = .0851) and the highest school grade achieved was 9.8 ± 2.1 and 10.5 ± 1.5 (*P* = .0237). Self-reported ethnic distribution was mixed ancestry 81% and 78%, black 13% and 15% and white 6% and 7% (*P* = .8549), being representative of the local community. For patients and controls respectively, the numbers of scans at each timepoint, were 83 and 96 at baseline, 39 and 52 at month 12 and 39 and 27 at month 24. Demographic, clinical, cognitive, and laboratory test details of the patients are provided in table 1. Neuroanatomical subtype assignment derived from the HYDRA results for the patients and controls at each timepoint is provided in Supplementary table 2.

**Table 1.** Demographic, Clinical, Cognitive and Laboratory Test Details of the Patients

	Mean	SD		
Age, years	24.66	6.8		
Highest school grade passed	9.85	2.1		
DUP (weeks)	37.12	47.8		
Modal antipsychotic dose (flupenthixol mg equivalents)	12.06	3.8		
% Adherence	98.46	3.7		
Weeks in study	68.82	39.5		
Childhood Trauma Questionnaire				
Total score	48.13	16.5		
Emotional abuse subscale	10.08	5.1		
Physical abuse subscale	9.82	5.3		
Sexual abuse subscale	7.25	4.1		
Emotional neglect subscale	12.26	5.4		
Physical neglect subscale	9.71	3.5		
Premorbid Adjustment Scale				
Childhood	0.24	0.2		
Early adolescence	0.29	0.2		
Late adolescence	0.37	0.2		
Adulthood	0.38	0.2		
General score	0.48	0.2		
Overall score	0.35	0.1		

	Baseline		M24	
	Mean	SD	Mean	SD
Baseline and M24 clinical, cognitive and laboratory assessments:				
PANSS Total score	93.02	14.9	42.28	10.6
PANSS Positive domain	17.52	3.4	5.35	2.8
PANSS Negative domain	19.20	5.4	9.35	4.1
PANSS Disorganized domain	11.98	3.0	5.37	2.0
ESRS Total score	1.13	3.0	2.06	5.2
MCCB Composite score	19.87	12.3	27.78	15.5
Speed of processing	22.14	11.9	28.24	13.0
Attention and vigilance	24.02	11.5	32.24	10.7
Working memory	23.88	14.2	31.65	11.9
Verbal learning	31.05	9.5	37.34	11.4
Visual learning	28.43	14.7	35.47	15.1
Reasoning and problem solving	30.17	9.0	38.77	12.5
Social cognition	40.80	15.8	40.80	21.6
BMI	21.83	4.1	25.03	5.3
Glucose	4.72	0.7	5.10	1.9
HDL-cholesterol	1.19	0.6	1.00	0.3
LDL-cholesterol	2.59	0.8	2.76	0.9
Triglycerides	0.90	0.5	1.12	0.9
Cholesterol	4.14	0.9	4.31	1.0

*Note:* DUP, duration of untreated psychosis; PANSS, Positive and Negative Syndrome Scale; ESRS, Extrapyramidal Symptom Rating Scale; MCCB, MATRICS Cognitive Consensus Battery; BMI, Body mass index; HDL, high-density lipoprotein; LDL, low-density lipoprotein.

### Signature Expression Strength Over the 2-Year Period for Patients and Controls

**Fig. 1.**
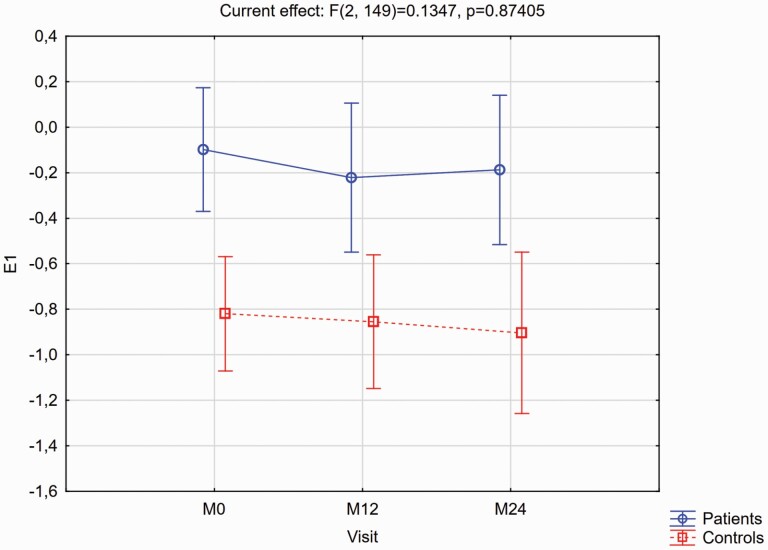
Expression of signature 1 (E1) for the patients v. controls, as visit-wise least square means and 95% confidence intervals from baseline to month 24, from the MMRM models. Mean (95% CI) signature 1 expression differences for patients vs controls at each timepoint, and for the within-group changes from baseline to M24 derived from the post hoc Fisher’s LSD test results were: Between group differences: M0 = 0.72 (0.35–1.09), *P* = .0002; M12 = 0.68 (0.11–1.26), *P* = .0049; M24 = 0.85 (0.21–1.50), *P* = .0094. Within-group differences from M0 to M24: Patients 0.12 (–0.26 to 0.50), *P* = .5302; Controls –0.01 (–0.44 to 0.43), *P* = .9819.

**Fig. 2.**
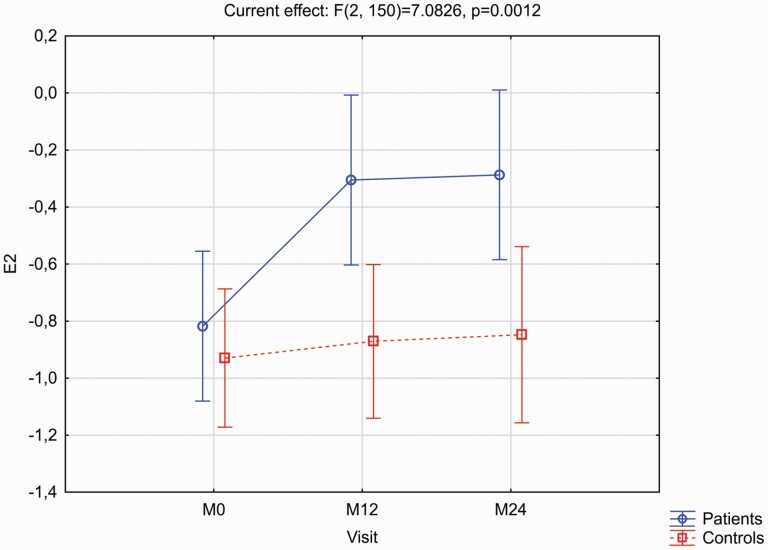
Expression of Signature 2 (E2) for the patients v. controls, as visit-wise least square means and 95% confidence intervals from baseline to month 24, from the MMRM models. Mean (95% CI) signature 2 expression differences for patients vs. controls at each timepoint, and for the within-group changes from baseline to M24 derived from the post hoc Fisher’s LSD test results were: Between group differences: M0 = 0.14 (–0.22 to 0.50), *P* = .4561; M12 = 0.59 (0.18–0.99), *P* = .0047; M24 = 0.58 (0.15–1.01), *P* = .0086. Within-group differences from M0 to M24: Patients –0.53 (–0.73 to 0.32), *P* ≤ .0001; Controls –0.08 (–0.32 to 0.15), *P* = .4926.

Figures 1 and 2 provide details of the expression strength of signatures 1 and 2 respectively over the two years in patients and controls, with the accompanying post hoc LSD test results as footnotes. For signature 1 the group*time interaction effect was not significant (*F* = 0.1347, *P* = .8741), and there were no significant within-group changes over time. However, at each timepoint patients had significantly greater signature 1 expression than controls. For signature 2 there was a significant group*time interaction (*F* = 6.9563, *P* = .0013). Expression strengths were similar between groups at baseline (*P* = .4561), and while controls did not change significantly over time (*P* = .4926) there was a highly significant increase in signature 2 strength in patients (*P* < .0001), with significant differences between the groups at M12 (*P* = .0047) and M24 (*P* = .0086).

### Neurodevelopmental Compromise and Signature Expression in Patients

Results of the fixed effect tests for our predictors of neurodevelopmental compromise on the two signatures, derived from the MMRM analyses, are provided in table 2. There were no significant effects for any of our selected predictors at the FDR corrected significance level in the primary analyses. Stronger signature 1 expression was associated at trend-level with lower educational attainment (*P* = .0608) and poorer NES total score (*P* = .0635). In the secondary analysis (table 3) stronger signature 1 expression was significantly associated (uncorrected) with poorer NES sensory integration subscale scores (*P* = .0256) and poorer performance in the cognitive domains of working memory (*P* = .0175), visual learning (*P* = .0430) and reasoning and problem solving (*P* = .0371). For signature 2 there were no significant or trend-level associations with any predictors of neurodevelopmental compromise in the primary analysis, and in the secondary analysis there was a trend-level association between greater signature expression and better premorbid adjustment in late adolescence (*P* = .053).

**Table 2.** Fixed Effect Test Results for the Primary Analysis of Potential Covariates, Predictors of Neurodevelopmental Compromise and Treatment Related Effects on the Two Signatures Expression Strengths, Derived from the MMRM Models

	Signature 1		Signature 2	
	*F*	*P* ^*^	*F*	*P* ^*^
Potential covariates				
Age	0.10	0.7532	1.22	0.2737
Gender	0.11	0.7422	0.01	0.9030
Ethnicity	0.15	0.7043	0.11	0.7381
DUP weeks	0.02	0.8756	0.22	0.6414
Axis 1 diagnosis	1.48	0.2283	0.43	0.5162
Cannabis number of positive tests	0.05	0.8176	2.30	0.1337
Factors associated with neurodevelopmental compromise				
Family history of schizophrenia	1.74	0.1912	0.26	0.6119
Obstetric complications	0.30	0.5870	0.39	0.5336
CTQ Total score	2.47	0.1217	0.11	0.7369
Highest grade passed	3.64	0.0608	1.08	0.3021
PAS Total General score	0.01	0.9199	2.30	0.1341
NES Total score	3.56	0.0635	0.05	0.8202
MCCB Composite score	0.88	0.3541	0.91	0.3459
Treatment related effects				
Flupenthixol dose	2.73	0.1029	0.00	0.9660
PANSS Total score	0.20	0.6564	20.32	<0.0001
ESRS Total score	0.19	0.6611	3.81	0.0550
BMI	0.30	0.5840	8.55	0.0046

*Note:* DUP, duration of untreated psychosis; CTQ, Childhood Trauma Questionnaire; PAS, Premorbid Adjustment Scale; NES, Neurological Evaluation Scale; MCCB, MATRICS Cognitive Consensus Battery; PANSS, Positive and Negative Syndrome Scale; ESRS, Extrapyramidal Symptom Rating Scale; BMI, Body mass index.

^*^FDR adjusted significance level = 0.0059.

**Table 3.** Fixed Effect Test Results for the Secondary Analysis of Potential Covariates, Predictors of Neurodevelopmental Compromise and Treatment Related Effects on the Two Signatures, Derived from the MMRM Models

	Subtype 1		Subtype 2	
	*F*	*P* ^*^	*F*	*P* ^*^
CTQ Subscale scores				
Emotional abuse	1.79	0.1869	0.07	0.7864
Physical abuse	0.89	0.3497	0.12	0.7266
Sexual abuse	1.75	0.1918	1.36	0.2488
Emotional neglect	0.70	0.4053	0.02	0.8814
Physical neglect	0.13	0.7181	0.23	0.6353
PAS Subscale scores				
Childhood	0.43	0.5158	0.04	0.8364
Early adolescence	2.82	0.0973	0.99	0.3219
Late adolescence	0.12	0.7289	3.87	0.0530
PANSS domain scores				
Positive	0.57	0.4536	20.22	<0.0001
Negative	0.17	0.6794	0.26	0.6147
Disorganized	1.41	0.2397	0.33	0.5660
NES Subscale scores				
Sensory Integration	5.19	0.0256	0.54	0.4643
Motor Coordination	1.81	0.1824	0.32	0.5757
Motor sequencing	0.71	0.4018	0.51	0.4770
MCCB cognitive domains				
Speed of processing	0.36	0.5497	0.41	0.5274
Attention and vigilance	0.62	0.4342	0.00	0.9988
Working memory	6.13	0.0175	0.02	0.9026
Verbal learning	0.00	0.9831	0.52	0.4739
Visual learning	4.36	0.0430	0.05	0.8161
Reasoning and problem solving	4.64	0.0371	0.46	0.5034
Social cognition	0.36	0.5553	0.47	0.4959
Fasting blood glucose and lipids				
Glucose	1.06	0.3061	2.21	0.1420
HDL-cholesterol	0.17	0.6846	0.38	0.5393
LDL-cholesterol	0.97	0.3280	0.04	0.8358
Triglycerides	0.08	0.7759	5.28	0.0245
Cholesterol	0.12	0.7322	0.86	0.3565

*Note:* CTQ, Childhood Trauma Questionnaire; PAS, Premorbid Adjustment Scale; PANSS, Positive and Negative Syndrome Scale; NES, Neurological Evaluation Scale; MCCB, MATRICS Cognitive Consensus Battery; HDL, high-density lipoprotein; LDL, low-density lipoprotein.

^*^P values uncorrected for multiple comparisons.

### Treatment Effects and Signature Expression in Patients

Increased signature 2 expression was significantly associated (corrected) with greater PANSS Total score reductions (*P* < .0001) and greater BMI increase (*P* = .0046), and at trend-level with ESRS Total score (*P* = .0550) (table 2). In the secondary analysis (table 3) increase in signature 2 expression was significantly associated (uncorrected) with greater symptom reduction in the PANSS positive domain (*P* < .0001), and increased triglycerides (*P* = .0245).

### Sensitivity Analysis

The analyses in the completers-only sample broadly confirmed those of the primary analyses. For the MMRM comparing signature 1 expression in patients versus controls the group*time interaction was non-significant (*F* = 1.3, *P* = .2683) but expression was significantly stronger (uncorrected) in the patients at baseline (*P* = .0022) and month 24 (*P* = .011) and at trend-level at month 12 (*P* = .0598). For signature 2 there was a near-significant group*time interaction (*F* = 3.1, *P* = .0510) and expression was similar in patients and controls at baseline (*P* = .8222) but increased significantly from baseline to month 12 (*P* = .0005) and month 24 (*P* < .0001) in patients, but not in controls at month 12 (*P* = .2471) and month 24 (0.4414). For the fixed effect tests in the completers-only sample, increase in signature 2 expression was significantly associated (uncorrected) with greater PANSS total score reductions (*P* = .0017) and with greater BMI increase (*P* = .0208).

## Discussion

In this study investigating the temporal stability and correlates of two recently described neuroanatomical signatures of schizophrenia in previously minimally treated individuals we found that the signatures displayed distinctly different trajectories over the 2-year treatment period and were differentially associated with indicators of neurodevelopmental compromise and treatment effects.

### Signature 1 and Neurodevelopmental Compromise

Several of our findings suggest a relationship between signature 1 and the neurodevelopmental component of schizophrenia. Consistent with the trait nature of neurodevelopmental deficits,^32^ signature 1 expression remained stable over time. However, this is at odds with previous reports of cortical thinning over time^33^ and our own earlier study finding of modest global cortical thickness reductions in patients but not controls.^9^ A possible confound here is that there may be some overlap between the signatures. Signature 1 includes some periventricular tissue, including thalamus and caudate, although these regions do not overlap with those of signature 2. The signature 1 score would then appear stable when in fact reductions in cortical thickness are balanced by increases in these structures in the basal ganglia. Furthermore, signature 1 was not influenced by treatment effects at all—again consistent with the proposal that subtype 1 represents non-dopaminergic abnormalities of the illness that are less responsive to DA blocking antipsychotics.^7^ However, we found only tentative links with our neurodevelopmental markers, suggesting weak effects for these variables and indicating caution when interpreting these results. Nevertheless, the trend-level association with poorer educational attainment is consistent with the initial study^7^ and the association with poorer cognitive performance was also reported in the second study.^8^ Finally, the trend-effect for neurological soft signs also suggests an association with underlying neurodevelopmental compromise. Subtle neurodevelopmental deficits including lower educational achievement, poorer cognitive ability and sensorimotor impairments are common in schizophrenia, and are considered promising candidates for endophenotypic markers of the illness.^34^ Unexpectedly, we did not find an association between negative symptom severity and signature 1 expression, as postulated by Crow. However, our negative symptom measure was not tailored for a defect state, and Crow specified that his type II syndrome related to those narrowly defined negative symptoms associated with the deficit state.^35^

### Signature 2 and Treatment Effects

Our most compelling findings relate to the associations between signature 2 and antipsychotic treatment. The strengthening of signature 2 expression in patients over the treatment period could reflect a general and global response to antipsychotics, consistent with increased striatal volumes observed in healthy rats treated with antipsychotics.^36^ Furthermore, that signature 2 expression was similar to the controls in the patients prior to receiving study treatment counts against this signature being directly related to the illness, at least in its pre-treatment neuroanatomic manifestations. But rather than a non-specific effect, the significant association between signature 2 expression strength and symptom reduction suggests a relationship to mechanisms underlying psychosis. The association was highly significant and specific to positive symptoms—the symptoms with the closest relation to DA dysfunction^37^ and for which antipsychotics work best.^38^ The relationship between signature 2 and treatment emergent weight gain provides an additional link with antipsychotic treatment, in this case an adverse effect. Similarly, the association with increased triglyceride levels in our secondary analysis suggests a relationship with deteriorating lipid profiles. Weight gain and accompanying dyslipidemia are common and serious side effects associated with most antipsychotics^39^ and importantly, have been linked to the clinical benefits of these agents,^40^ thereby raising the possibility of a shared underlying mechanistic pathway.^41^ It is possible that signature 2 defines this mechanistic pathway. Finally, the trend-level association with ESRS scores suggests that signature 2 may also be related to antipsychotic induced motor symptoms.

The most likely mechanism underlying these associations would be via DA pathways, given that antipsychotic efficacy as well as treatment emergent extrapyramidal symptoms and (at least in part) antipsychotic induced weight gain have all been attributed to the DA receptor antagonistic effects of these agents.^42^ However, notwithstanding our ability to assess the dose with precision, we did not find a relationship between antipsychotic dose and signature 2 expression, consistent with a recent report of a hyperbolic dose-response with flattening of the curve beyond a certain point.^43^

Relating structural MRI measures to underlying cellular pathology is challenging. MRI is not a direct measure of brain structure and may be confounded by epiphenomena and artifacts.^44^ Nevertheless, several mechanisms could explain the link between antipsychotic responsiveness and increases in signature 2 expression and basal ganglia volume. While antipsychotics are proposed to have neuroprotective effects ranging from preventive to restorative mediated via multiple mechanisms including neurogenesis,^45^ counting against this is that baseline measures were similar in patients and controls and the treatment-associated increases in patients went beyond those of the healthy controls. This rather suggests an adaptive or compensatory response, possibly involving structural remodeling, changes in water content,^46^ microglial proliferation,^47^ or augmented blood flow to the striatum.^48^

Nonetheless, a treatment effect cannot fully explain the occurrence of signature 2, particularly when the neuroanatomical subtypes are considered categorically. Although not part of our analyses and only presented as Supplementary material, when allocated to subtypes,^7^ 22% of our patients were classified as subtype 2 (either alone or together with subtype 1) prior to treatment. Subtype 2 was also present in 22% of our healthy controls. Indeed, the presence of both subtypes in some controls aligns with previous findings in healthy adults and may represent a biological vulnerability to schizophrenia found in the general population, the majority of whom will never develop the illness.^8^ Also, these supplementary findings suggest that pre-treatment allocation to subtypes does not predict treatment outcomes or changes in cortical thickness, basal ganglia or white matter volumes (Supplementary table 3).

### Study Strengths and Weaknesses

Study strengths are related to the unique nature of our sample that allowed us to investigate the neuroanatomical signatures prior to treatment and to assess their trajectories longitudinally compared to healthy volunteers. We were also able to comprehensively assess treatment related effects in terms of the precise antipsychotic dose received, efficacy, and adverse effects. A further strength is that our sample was completely independent of the discovery sample, thereby providing additional information on the signatures in individuals with schizophrenia. There are also study limitations. First, while the sample size is relatively large for a single-site study of this nature the study power is limited when compared to larger multisite samples. This limitation needs to be weighed against the advantages of a single-site study such as standardized treatment, uniformity of assessments and the use of a single MRI scanner. Second, as with most longitudinal studies in schizophrenia^49^ the attrition rate was considerable. While the MMRM models offer a powerful framework for analyzing longitudinal imaging data^50^ the missing values introduce a risk of bias. However, counting against this is that our sensitivity analysis with completers-only produced a similar pattern to our main findings. Third, while our use of a single antipsychotic avoided possible confounding treatment effects of multiple antipsychotics, it also limits generalization of our findings to other antipsychotics with different pharmacologic profiles. Fourth, associations between neurodevelopmental compromise and signature 1 expression were largely non-significant and must be interpreted with caution. Finally, longer term changes in subtype signature expression cannot be extrapolated from our findings and would need to be evaluated in studies conducted over a longer study period.

In conclusion, we provide evidence that two distinct neuroanatomical signatures represent treatment-responsive and non-responsive components of the illness that may be linked to dopaminergic and neurodevelopmental components of schizophrenia. Future studies should include larger samples, a longer study period and additional indicators of neurodevelopmental deviance.

## Supplementary Material

sbad040_suppl_Supplementary_Table_S1

sbad040_suppl_Supplementary_Table_S2

sbad040_suppl_Supplementary_Table_S3

sbad040_suppl_Supplementary_Table_S4

sbad040_suppl_Supplementary_Table_S5

sbad040_suppl_Supplementary_Material
