# Supplementary material for: Two Neuroanatomical Signatures in Schizophrenia: Expression Strengths Over the First 2 Years of Treatment and Their Relationships to Neurodevelopmental Compromise and Antipsychotic Treatment
Source: Schizophr Bull. 2023 Apr 12;49(4):1067–77. doi: 10.1093/schbul/sbad040 (PMC10318886; doi:10.1093/schbul/sbad040)
Supplement: sbad040_suppl_Supplementary_Table_S1 [file sbad040_suppl_supplementary_table_s1.docx]

**Supplementary Table 1.** MUSE regions of interest used to derive SZ signature expressions. T1 was segmented into 145 anatomical regions of interest from the grey matter, white matter, and cerebrospinal fluid volumes

| 3rd ventricle | Ventral diencephalon (R) | Anterior insula (L) |
| --- | --- | --- |
| 4th ventricle | Ventral diencephalon (L) | Anterior orbital gyrus (R) |
| Accumbens area (R) | Cerebellar vermal lobules I-V | Anterior orbital gyrus (L) |
| Accumbens area (L) | Cerebellar vermal lobules VI-VII | Angular gyrus (R) |
| Amygdala (R) | Cerebellar vermal lobules VIII-X | Angular gyrus (L) |
| Amygdala (L) | Basal forebrain (R) | Calcarine cortex (R) |
| Brain Stem | Basal forebrain (L) | Calcarine cortex (L) |
| Caudate (R) | Frontal lobe WM (R) | Central operculum (R) |
| Caudate (L) | Frontal lobe WM (L) | Central operculum (L) |
| Cerebellum exterior (R) | Occipital lobe WM (R) | Cuneus (R) |
| Cerebellum exterior (L) | Occipital lobe WM (R) | Cuneus (L) |
| Cerebellum WM (R) | Parietal lobe WM (R) | Entorhinal area (R) |
| Cerebellum WM (L) | Parietal lobe WM (L) | Entorhinal area (L) |
| Hippocampus (R) | Temporal lobe WM (R) | Frontal operculum (R) |
| Hippocampus (L) | Temporal lobe WM (L) | Frontal operculum (L) |
| Inferior lateral ventricle (R) | Fornix (R) | Frontal pole (R) |
| Inferior lateral ventricle (L) | Fornix (L) | Frontal pole (L) |
| Lateral ventricle (R) | Anterior limb of internal capsule (R) | Fusiform gyrus (R) |
| Lateral ventricle (L) | Anterior limb of internal capsule (L) | Fusiform gyrus (L) |
| Pallidum (R) | Posterior limb of internal capsule including cerebral peduncle (R) | Gyrus rectus (R) |
| Pallidum (L) | Posterior limb of internal capsule including cerebral peduncle (L) | Gyrus rectus (L) |
|  |  |  |
| Putamen (R) |  | Inferior occipital gyrus (R) |
| Putamen (L) | Corpus callosum | Inferior occipital gyrus (L) |
| Thalamus proper (R) | Anterior cingulate gyrus (R) | Inferior temporal gyrus (R) |
| Thalamus proper (L) | Anterior cingulate gyrus (L) | Inferior temporal gyrus (L) |
| Lingual gyrus (R) | Anterior insula (R) | Subcallosal area (R) |
| Lingual gyrus (L) | Occipital fusiform gyrus (L) | Subcallosal area (L) |
| Lateral orbital gyrus (R) | Opercular part of inferior frontal gyrus (R) | Superior frontal gyrus (R) |
| Lateral orbital gyrus (L) | Opercular part of inferior frontal gyrus (L) | Superior frontal gyrus (L) |
| Middle cingulate gyrus (R) | Orbital part of inferior frontal gyrus (R) | Supplementary motor cortex (R) |
| Middle cingulate gyrus (L) | Orbital part of inferior frontal gyrus (L) | Supplementary motor cortex (L) |
| Medial frontal cortex (R) | Posterior cingulate gyrus (R) | Supramarginal gyrus (R) |
| Medial frontal cortex (L) | Posterior cingulate gyrus (L) | Supramarginal gyrus (L) |
| Middle frontal gyrus (R) | Precuneus (R) | Superior occipital gyrus (R) |
| Middle frontal gyrus (L) | Precuneus (L) | Superior occipital gyrus (L) |
| Middle occipital gyrus (R) | Parahippocampal gyrus (R) | Superior parietal lobule (R) |
| Middle occipital gyrus (L) | Parahippocampal gyrus (L) | Superior parietal lobule (L) |
| Medial orbital gyrus (R) | Posterior insula (R) | Superior temporal gyrus (R) |
| Medial orbital gyrus (L) | Posterior insula (L) | Superior temporal gyrus (L) |
| Postcentral gyrus medial segment (R) | Parietal operculum (R) | Temporal pole (R) |
| Postcentral gyrus medial segment (L) | Parietal operculum (L) | Temporal pole (L) |
| Precentral gyrus medial segment (R) | Postcentral gyrus (R) | Triangular part of the inferior frontal gyrus (R) |
| Precentral gyrus medial segment (L) | Postcentral gyrus (L) |  |
| Superior frontal gyrus medial segment (R) | Posterior orbital gyrus (R) | Triangular part of the inferior frontal gyrus (L) |
|  | Posterior orbital gyrus (L) |  |
| Superior frontal gyrus medial segment (L) | Planum polare (R) | Transverse temporal gyrus (R) |
| Middle temporal gyrus (R) | Planum polare (L) | Transverse temporal gyrus (L) |
| Middle temporal gyrus (L) | Precentral gyrus (R) |  |
| Occipital pole (R) | Precentral gyrus (L) |  |
| Occipital pole (L) | Planum temporale (R) |  |
| Occipital fusiform gyrus (R) | Planum temporale (L) |  |

L, Left Hemisphere; R, Right Hemisphere; WM, White matter
