# Supplementary material for: Two Neuroanatomical Signatures in Schizophrenia: Expression Strengths Over the First 2 Years of Treatment and Their Relationships to Neurodevelopmental Compromise and Antipsychotic Treatment
Source: Schizophr Bull. 2023 Apr 12;49(4):1067–77. doi: 10.1093/schbul/sbad040 (PMC10318886; doi:10.1093/schbul/sbad040)
Supplement: sbad040_suppl_Supplementary_Table_S2 [file sbad040_suppl_supplementary_table_s2.docx]

**Supplementary Table 2.** Subtype assignment derived from the HYDRA results for the patients and controls at each timepoint.

| Timepoint | Group | Subtype | | | |
| --- | --- | --- | --- | --- | --- |
|  |  | S1 | S2 | S1+S2 | S0 |
| Baseline | Patients | 29 (35%) | 14 (17%) | 4 (5%) | 36(43 %) |
|  | Controls | 19 (20%) | 19 (20%) | 2 (2%) | 56 (58%) |
| Month 12 | Patients | 12 (31%) | 11 (28%) | 5 (13 %) | 11 (28%) |
|  | Controls | 13 (25%) | 10 (19%) | 4 (8%) | 25 (48%) |
| Month 24 | Patients | 11 (28%) | 9 (23%) | 7 (18%) | 12 (31%) |
|  | Controls | 5 (19%) | 9 (33%) | 0 (0%) | 13 (48%) |
