# Supplementary material for: Two Neuroanatomical Signatures in Schizophrenia: Expression Strengths Over the First 2 Years of Treatment and Their Relationships to Neurodevelopmental Compromise and Antipsychotic Treatment
Source: Schizophr Bull. 2023 Apr 12;49(4):1067–77. doi: 10.1093/schbul/sbad040 (PMC10318886; doi:10.1093/schbul/sbad040)
Supplement: sbad040_suppl_Supplementary_Table_S3 [file sbad040_suppl_supplementary_table_s3.docx]

**Supplementary Table 3.** Demographic and clinical details for the patients according to baseline neuroanatomical signature assignment.

|  | **Signature 1**  **n=29** | **Signature 2**  **n=14** | **Signatures 1+2**  **n=4** | **Signature 0**  **n=36** | **Test value and P^1^** |
| --- | --- | --- | --- | --- | --- |
| Age, years, median (IQR) | 22 (19 to 26) | 23 (20 to 27) | 24 (22 to 25) | 24 (20 to 28) | 1.48, p=0.6876 |
| Males, n (%) | 21 (72%) | 9 (64%) | 3 (75%) | 28 (78%) | 1.35, p=0.7171 |
| Highest school grade attained, years, median (IQR) | 9 (8 to 10) | 11 (9 to 12) | 9 (6 to 12) | 12 (9 to 12) | 7.75, p=0.0514**^3^** |
| Premorbid Adjustment Scale Overall score, median (IQR) | 0.33 (0.24 to 0.44) | 0.29 (0.18 to 0.37) | 0.32 (0.26 to 0.49) | 0.27 (0.21 to 0.40) | 2.44, p=0.4854 |
| Recent substance use,^2^ n (%) | 17 (61%) | 9 (64%) | 3 (75%) | 23 (64%) | 0.33, p=0.9548 |
| Age of onset of psychosis, years, median (IQR) | 20.80 (18.98 to 24.36) | 22.84 (19.74 to 26.91) | 23.20 (21.30 to 24.18) | 23.63 (19.76 to 27.92) | 1.77, p=0.6205 |
| Duration of untreated psychosis, weeks), median (IQR) | 26.00 (9.58 to 54.58) | 13.93 (7.15 to 39.15) | 12.87 (10.44 to 16.51) | 22.22 (10.08 to 40.08) | 1.67, p=0.6444 |
| Antipsychotic naïve at study entry, n (%) | 16 (57%) | 8 (62%) | 1 (25%) | 21 (58%) | 1.80, p=0.6143 |
| Hospitalised during the study, n (%) | 19 (66%) | 6 (43%) | 3 (75%) | 22 (61%) | 2.48, p=0.4792 |
| Modal study antipsychotic dose (flupenthixol mg equiv), median (IQR) | 10 (10 to 15) | 10 (10 to 15) | 10 (8 to 16) | 10 (10 to 15) | 0.90, p=0.8250 |
| Cumulative study antipsychotic dose (flupenthixol mg equiv), median (IQR) | 1740 (917 to 2139) | 1715 (1267 to 2160) | 480 (138 to 2090) | 1355 (536 to 1864) | 2.26, p=0.5207 |
| MCCB Composite score M0, median (IQR) | 19 (10 to 25) | 14 (5 to 27) | 27 (27 to 27) | 16 (11 to 32) | 1.43, p=0.6987 |
| MCCB Composite score change, median (IQR) | 2 (-3 to 5) | 17 (7 to 26) | 16 (16 to 16) | 9 (3 to 18) | 7.30, p=0.0629 |
| PANSS Total M0, median (IQR) | 90 (83 to 104) | 97 (88 to 104) | 103 (94 to 108) | 93 (82 to 99) | 4.01, p=0.2599 |
|  |  |  |  |  |  |
| PANSS Total change, median (IQR) | -38 (-58 to 28) | -47 (-64 to -12) | -30 (-55 to -22) | -42 (-50 to -30) | 0.31, , p=0.9573 |
| PANSS Positive factor M0, median (IQR) | 19 (15 to 20) | 17.5 (15 to 20) | 19 (19 to 20) | 18 (16 to 20) | 1.43, p=0,6977 |
| PANSS Positive factor change, median (IQR) | -11 (-15 to-6) | -10 (-15 to -5) | -7 (-13 to -4) | -11 (-14 to -6) | 0.97, p=0.8088 |
| PANSS Negative factor M0, median (IQR) | 20 (15 to 23) | 20 (17 to 22) | 23 (18 to 23) | 20 (16 to 23) | 0.71, p=0.8691 |
| PANSS Negative factor change, median (IQR) | -8.5 (-14.5 to -5) | -9 (-15 to -3) | -7 (-14 to -6) | -10 (-12 to -6) | 0.28, p=0.9643 |
| PANSS Disorganized factor M0, median (IQR) | 12 (11 to 14) | 12 (10 to 14) | 15 (13 to 16) | 11 (9 to 14) | 3.87, p=0.2753 |
| PANSS Disorganized factor change, median (IQR) | -6 (-7 to -4) | -5 (-7 to -2) | -5 (-10 to -3) | -5 (-7 to -3) | 1.69, p=0.6388 |
| Remission at endpoint, n (%) | 13 (45%) | 9 (64%) | 1 (25%) | 24 (67%) | 4.63, p=0.2011 |
| Relapse during the study, n (%) | 5 (17%) | 2 (14%) | 1 (25%) | 1 (3%) | 4.65, p=0.1991 |
| White matter volume M0, median (IQR) | 31.26 (29.41 to 33.93) | 31.50 (30.14 to 32.72) | 31.08 (30.79 to 32.31) | 32.44 (30.06 to 35.13) | 2.62, p=0.4548 |
| White matter volume change, median (IQR) | 1.48 (0.23 to 2.66) | -0.02 (-0.94 to 1.52) | -0.19 (-0.19 to 0.19) | 0.88 (-0.40 to 2.48) | 3.75, p=0.2893 |
| Basal ganglia volume M0, median (IQR) | 1.41 (1.33 to 1.49) | 1.43 (1.36 to 1.53) | 1.42 (1.35 to 1.48) | 1.33 (1.25 to 1.43) | 8.09, p=0.0441^4^ |
| Basal ganglia volume change, median (IQR) | 0.05 (-0.03 to 0.12) | 0.00 (-0.10 to 0.03) | 0.05 (0.05 to 0.05) | 0.02 (-0.03 to 0.08) | 2.18, p=0.5354 |
| Global cortical thickness M0, median (IQR) | 2.42 (2.37 to 2.48) | 2.51 (2.35 to 2.54) | 2.24 (2.15 to 2.41) | 2.42 (2.36 to 2.53) | 4.61, p=0.2026 |
| Global cortical thickness change , median (IQR) | -0.07 (-0.15 to -0.01) | -0.02 (-0.06 to 0.05) | 0.06 (0.06 to 0.06 | -0.05 (-0.10 to -0.02) | 3.19, p=0.3629 |
|  |  |  |  |  |  |
| ^1^χ2 and H for Chi square and Kruskal Wallis respectively  ^2^Recent substance use defined as in the 3 months prior to study entry.  ^3^Mann-Whitney U Test indicates signature1 differs significantly from signature 0, p=0.0151  ^4^Mann-Whitney U Test indicates significant differences between singature1 and signature 0 (p=0.0320) and between signature 2 and signature 0 (p=0.0191)  IQR = interquartile range, MCCB = MATRICS Cognitive Consensus Battery, PANSS = Positive and negative syndrome scale, M0=baseline, change=change from baseline to endpoint value | | | | | |
