# Supplementary material for: Two Neuroanatomical Signatures in Schizophrenia: Expression Strengths Over the First 2 Years of Treatment and Their Relationships to Neurodevelopmental Compromise and Antipsychotic Treatment
Source: Schizophr Bull. 2023 Apr 12;49(4):1067–77. doi: 10.1093/schbul/sbad040 (PMC10318886; doi:10.1093/schbul/sbad040)
Supplement: sbad040_suppl_Supplementary_Table_S4 [file sbad040_suppl_supplementary_table_s4.docx]

**Supplementary Table 4.** Fixed effect test results for the primary MMRM analyses with signature expression strength as the dependent variable

|  | | | | | | | | | |
| --- | --- | --- | --- | --- | --- | --- | --- | --- | --- |
|  | **Signature 1** | | | |  | **Signature 2** | | | |
| **Patients vs controls with Signatures 1 and 2 as the dependent variables:** | | | | | | | | | |
|  | Num. DF | Den. DF | F | p |  | Num. DF | Den. DF | F | p |
| Age | 1 | 149 | 0.07 | 0.7900 |  | 1 | 150 | 0.17 | 0.6820 |
| Gender | 1 | 149 | 2.67 | 0.1046 |  | 1 | 150 | 0.00 | 0.9769 |
| Group (Patient vs control) | 1 | 149 | 13.81 | 0.0003 |  | 1 | 150 | 5.20 | 0.0240 |
| Visit | 2 | 149 | 0.54 | 0.5811 |  | 2 | 150 | 11.99 | <0.0001 |
| Visit*Group | 2 | 149 | 0.13 | 0.8741 |  | 2 | 150 | 7.08 | 0.0012 |
|  |  |  |  |  |  |  |  |  |  |
| **Patients only with Signatures 1 and 2 as the dependent variables:** | | | | | | | | | |
|  | Num. DF | Den. DF | F | p |  | Num. DF | Den. DF | F | p |
| Age | 1 | 70 | 0.10 | 0.7532 |  | 1 | 70 | 1.22 | 0.2737 |
| Gender | 1 | 70 | 0.11 | 0.7422 |  | 1 | 70 | 0.01 | 0.9030 |
| Ethnicity | 1 | 70 | 0.15 | 0.7043 |  | 1 | 70 | 0.11 | 0.7381 |
| DUP. weeks | 1 | 70 | 0.02 | 0.8756 |  | 1 | 70 | 0.22 | 0.6414 |
| Axis 1 diagnosis | 1 | 70 | 1.48 | 0.2283 |  | 1 | 70 | 0.43 | 0.5162 |
| Cannabis number of positive tests | 1 | 70 | 0.05 | 0.8176 |  | 1 | 70 | 2.30 | 0.1337 |
| Visit | 2 | 70 | 0.73 | 0.4844 |  | 2 | 70 | 16.34 | <0.0001 |
|  |  |  |  |  |  |  |  |  |  |
| Family history of Schizophrenia | 1 | 67 | 1.74 | 0.1912 |  | 1 | 67 | 0.26 | 0.6119 |
| Obstetric complications | 1 | 67 | 0.30 | 0.5870 |  | 1 | 67 | 0.39 | 0.5336 |
| CTQ Total score | 1 | 56 | 2.47 | 0.1217 |  | 1 | 56 | 0.11 | 0.7369 |
| Highest grade passed | 1 | 67 | 3.64 | 0.0608 |  | 1 | 67 | 1.08 | 0.3021 |
| PAS Total General score | 1 | 67 | 0.01 | 0.9199 |  | 1 | 67 | 2.30 | 0.1341 |
| NES Total score | 1 | 67 | 3.56 | 0.0635 |  | 1 | 67 | 0.05 | 0.8202 |
| MCCB Composite score | 1 | 33 | 0.88 | 0.3541 |  | 1 | 33 | 0.91 | 0.3459 |
| Visit | 2 | 67 | 0.26 | 0.7741 |  | 2 | 67 | 19.85 | <0.0001 |
|  |  |  |  |  |  |  |  |  |  |
| Flupenthixol dose | 1 | 71 | 2.73 | 0.1029 |  | 1 | 71 | 0.00 | 0.9660 |
| PANSS Total score | 1 | 71 | 0.20 | 0.6564 |  | 1 | 71 | 20.32 | <0.0001 |
| ESRS Total score | 1 | 71 | 0.19 | 0.6611 |  | 1 | 71 | 3.81 | 0.0550 |
| BMI | 1 | 71 | 0.30 | 0.5840 |  | 1 | 71 | 8.55 | 0.0046 |
| Visit | 2 | 71 | 0.14 | 0.8704 |  | 2 | 71 | 1.93 | 0.1521 |

Num. DF = numerator degrees of freedom; Den. DF = denominator degrees of freedom; DUP = Duration of untreated psychosis; CTQ = Childhood trauma questionnaire; PAS = Premorbid adjustment scale; NES = Neurological evaluation scale; MCCB = MATRICS Cognitive Consensus Battery; PANSS = Positive and negative syndrome scale; ESRS = Extrapyramidal Symptom Rating Scale; BMI = Body mass index.

*FDR adjusted significance level = 0.0059
