# Supplementary material for: Two Neuroanatomical Signatures in Schizophrenia: Expression Strengths Over the First 2 Years of Treatment and Their Relationships to Neurodevelopmental Compromise and Antipsychotic Treatment
Source: Schizophr Bull. 2023 Apr 12;49(4):1067–77. doi: 10.1093/schbul/sbad040 (PMC10318886; doi:10.1093/schbul/sbad040)
Supplement: sbad040_suppl_Supplementary_Table_S5 [file sbad040_suppl_supplementary_table_s5.docx]

**Supplementary Table 5.** Post-hoc inter- and intra-group comparisons of signature expression strengths derived from the MMRM models.

| Signature 1 mean ± CI difference and Fishers Least Significant Difference for patients and controls at each of the timepoints: | | | | |
| --- | --- | --- | --- | --- |
|  | Mean difference | -95,00% | 95,00% | p |
| Patients M0 vs Controls M0 | 0,72 | 0,35 | 1,09 | 0,0002 |
| Patients M0 vs Patients M12 | 0,12 | -0,14 | 0,39 | 0,3604 |
| Patients M0 vs Patients M24 | 0,09 | -0,18 | 0,36 | 0,5065 |
| Controls M0 vs Controls M12 | 0,03 | -0,20 | 0,27 | 0,7736 |
| Controls M0 vs Controls M24 | 0,08 | -0,22 | 0,39 | 0,5914 |
| Patients M12 vs Controls M12 | 0,63 | 0,19 | 1,07 | 0,0052 |
| Patients M12 vs Patients M24 | -0,03 | -0,33 | 0,26 | 0,8219 |
| Controls M12 vs Controls M24 | 0,05 | -0,27 | 0,37 | 0,7596 |
| Patients M24 vs Controls M24 | 0,72 | 0,23 | 1,20 | 0,0040 |
|  |  |  |  |  |
| Signature 2 mean ±CI difference and Fishers Least Significant Difference for patients and controls at each of the timepoints: | | | | |
|  | Mean | -95,00% | 95,00% | p |
| Patients M0 vs Controls M0 | 0,11 | -0,25 | 0,47 | 0,5394 |
| Patients M0 vs Patients M12 | -0,51 | -0,72 | -0,31 | <0,0001 |
| Patients M0 vs Patients M24 | -0,53 | -0,73 | -0,33 | <0,0001 |
| Controls M0 vs Controls M12 | -0,06 | -0,24 | 0,12 | 0,5190 |
| Controls M0 vs Controls M24 | -0,08 | -0,32 | 0,15 | 0,4904 |
| Patients M12 vs Controls M12 | 0,57 | 0,16 | 0,97 | 0,0062 |
| Patients M12 vs Patients M24 | -0,02 | -0,24 | 0,21 | 0,8731 |
| Controls M12 vs Controls M24 | -0,02 | -0,26 | 0,22 | 0,8478 |
| Patients M24 vs Controls M24 | 0,56 | 0,13 | 0,99 | 0,0110 |
