# Supplementary material for: Two Neuroanatomical Signatures in Schizophrenia: Expression Strengths Over the First 2 Years of Treatment and Their Relationships to Neurodevelopmental Compromise and Antipsychotic Treatment
Source: Schizophr Bull. 2023 Apr 12;49(4):1067–77. doi: 10.1093/schbul/sbad040 (PMC10318886; doi:10.1093/schbul/sbad040)
Supplement: sbad040_suppl_Supplementary_Material [file sbad040_suppl_supplementary_material.docx]

APPENDIX A.

Publications arising from the study cohort to date:

1: Luckhoff HK, Asmal L, Scheffler F, du Plessis S, Chiliza B, Smit R,

Phahladira L, Emsley R. Sex and gender associations with indicators of

neurodevelopmental compromise in schizophrenia spectrum disorders. Schizophr

Res. 2022 May;243:70-77. doi: 10.1016/j.schres.2022.02.012. Epub 2022 Mar 1.

PMID: 35245704.

2: Phahladira L, Asmal L, Lückhoff HK, du Plessis S, Scheffler F, Smit R,

Chiliza B, Emsley R. The trajectories and correlates of two negative symptom

subdomains in first-episode schizophrenia. Schizophr Res. 2022 May;243:17-23.

doi: 10.1016/j.schres.2022.02.017. Epub 2022 Feb 25. PMID: 35228035.

3: Emsley R, du Plessis S, Phahladira L, Luckhoff HK, Scheffler F, Kilian S,

Smit R, Buckle C, Chiliza B, Asmal L. Antipsychotic treatment effects and

structural MRI brain changes in schizophrenia. Psychol Med. 2021 Sep 23:1-10.

doi: 10.1017/S0033291721003809. Epub ahead of print. PMID: 35441587.

4: Luckhoff HK, Asmal L, Scheffler F, du Plessis S, Buckle C, Chiliza B, Kilian

S, Smit R, Phahladira L, Emsley R. Gender role endorsement in first-episode

schizophrenia spectrum disorders. Psychiatry Res. 2021 May;299:113867. doi:

10.1016/j.psychres.2021.113867. Epub 2021 Mar 9. PMID: 33751988.

5: Phahladira L, Asmal L, Lückhoff HK, du Plessis S, Scheffler F, Kilian S, Smit

R, Buckle C, Chiliza B, Emsley R. The course and concomitants of depression in

first-episode schizophrenia spectrum disorders: A 24-month longitudinal study.

Psychiatry Res. 2021 Apr;298:113767. doi: 10.1016/j.psychres.2021.113767. Epub

2021 Jan 29. PMID: 33545422.

6: Benelmokhtar JM, Chiliza B, Phahladira L, Emsley R, Asmal L. Factors

associated with dropout at 2 years post-initiation of treatment in the first

episode of schizophrenia. S Afr J Psychiatr. 2021 Mar 9;27:1657. doi:

10.4102/sajpsychiatry.v27i0.1657. PMID: 33824760; PMCID: PMC8008008.

7: Joubert FP, Chiliza B, Emsley R, Asmal L. Extrapyramidal side effects in

first-episode schizophrenia treated with flupenthixol decanoate. S Afr J

Psychiatr. 2021 Jan 11;27:1568. doi: 10.4102/sajpsychiatry.v27i0.1568.

8: Luckhoff HK, du Plessis S, Scheffler F, Phahladira L, Kilian S, Buckle C,

Smit R, Chiliza B, Asmal L, Emsley R. Fronto-limbic white matter fractional

anisotropy and body mass index in first-episode schizophrenia spectrum disorder

patients compared to healthy controls. Psychiatry Res Neuroimaging. 2020 Nov

30;305:111173. doi: 10.1016/j.pscychresns.2020.111173. Epub 2020 Sep 2. PMID:

32896691.

9: Luckhoff H, Asmal L, Kilian S, Scheffler F, du Plessis S, Buckle C,

Phahladira L, Smit R, Chiliza B, Emsley R. Sex versus gender as risk factors for

the age of onset of schizophrenia spectrum disorders. Schizophr Res. 2020

Jun;220:267-268. doi: 10.1016/j.schres.2020.04.012. Epub 2020 Apr 21. PMID:

32327315.

10: Phahladira L, Luckhoff HK, Asmal L, Kilian S, Scheffler F, Plessis SD,

Chiliza B, Emsley R. Early recovery in the first 24 months of treatment in

first-episode schizophrenia-spectrum disorders. NPJ Schizophr. 2020 Jan

8;6(1):2. doi: 10.1038/s41537-019-0091-y. PMID: 31913311; PMCID: PMC6949247.

11: Retief M, Chiliza B, Phahladira L, Emsley R, Asmal L. Prolactin,

flupenthixol decanoate and first episode schizophrenia - clinical and laboratory

correlates. Metab Brain Dis. 2019 Dec;34(6):1679-1687. doi:

10.1007/s11011-019-00474-5. Epub 2019 Aug 17. PMID: 31422510.

12: Luckhoff HK, Kilian S, Olivier MR, Phahladira L, Scheffler F, du Plessis S,

Chiliza B, Asmal L, Emsley R. Relationship between changes in metabolic syndrome

constituent components over 12 months of treatment and cognitive performance in

first-episode schizophrenia. Metab Brain Dis. 2019 Apr;34(2):469-476. doi:

10.1007/s11011-018-0372-6. Epub 2019 Jan 2. PMID: 30604027.

13: Luckhoff H, Phahladira L, Scheffler F, Asmal L, du Plessis S, Chiliza B,

Kilian S, Emsley R. Weight gain and metabolic change as predictors of symptom

improvement in first-episode schizophrenia spectrum disorder patients treated

over 12 months. Schizophr Res. 2019 Apr;206:171-176. doi:

10.1016/j.schres.2018.11.031. Epub 2018 Nov 30. PMID: 30503765.

14: Phahladira L, Asmal L, Kilian S, Chiliza B, Scheffler F, Luckhoff HK, du

Plessis S, Emsley R. Changes in insight over the first 24 months of treatment in

schizophrenia spectrum disorders. Schizophr Res. 2019 Apr;206:394-399. doi:

10.1016/j.schres.2018.10.013. Epub 2018 Oct 29. PMID: 30385130.

15: Asmal L, Kilian S, du Plessis S, Scheffler F, Chiliza B, Fouche JP, Seedat

S, Dazzan P, Emsley R. Childhood Trauma Associated White Matter Abnormalities in

First-Episode Schizophrenia. Schizophr Bull. 2019 Mar 7;45(2):369-376. doi:

10.1093/schbul/sby062. PMID: 29860345; PMCID: PMC6403087.

16: Kilian S, Asmal L, Chiliza B, Olivier MR, Phahladira L, Scheffler F, Seedat

S, Marder SR, Green MF, Emsley R. Childhood adversity and cognitive function in

schizophrenia spectrum disorders and healthy controls: evidence for an

association between neglect and social cognition. Psychol Med. 2018

Oct;48(13):2186-2193. doi: 10.1017/S0033291717003671. Epub 2017 Dec 22. PMID:

29268811.

17: Scheffler F, Kilian S, Chiliza B, Asmal L, Phahladira L, du Plessis S, Kidd

M, Murray RM, Di Forti M, Seedat S, Emsley R. Effects of cannabis use on body

mass, fasting glucose and lipids during the first 12 months of treatment in

schizophrenia spectrum disorders. Schizophr Res. 2018 Sep;199:90-95. doi:

10.1016/j.schres.2018.02.050. Epub 2018 Mar 6. PMID: 29519756.

18: Asmal L, du Plessis S, Vink M, Chiliza B, Kilian S, Emsley R. Symptom

attribution and frontal cortical thickness in first-episode schizophrenia. Early

Interv Psychiatry. 2018 Aug;12(4):652-659. doi: 10.1111/eip.12358. Epub 2016 Aug

29. PMID: 27572938.

19: Emsley R, Chiliza B, Asmal L, Kilian S, Riaan Olivier M, Phahladira L,

Ojagbemi A, Scheffler F, Carr J, Kidd M, Dazzan P. Neurological soft signs in

first-episode schizophrenia: State- and trait-related relationships to

psychopathology, cognition and antipsychotic medication effects. Schizophr Res.

2017 Oct;188:144-150. doi: 10.1016/j.schres.2017.01.034. Epub 2017 Jan 24. PMID:

28130002.

20: Emsley R, Asmal L, du Plessis S, Chiliza B, Phahladira L, Kilian S. Brain

volume changes over the first year of treatment in schizophrenia: relationships

to antipsychotic treatment. Psychol Med. 2017 Sep;47(12):2187-2196. doi:

10.1017/S0033291717000642. Epub 2017 Mar 28. PMID: 28347393.

21: Olivier RM, Kilian S, Chiliza B, Asmal L, Oosthuizen PP, Emsley R, Kidd M.

Cognitive-perceptual deficits and symptom correlates in first-episode

schizophrenia. S Afr J Psychiatr. 2017 Aug 31;23:1049. doi:

10.4102/sajpsychiatry.v23i0.1049. PMID: 30263189; PMCID: PMC6138160.

22: Asmal L, du Plessis S, Vink M, Fouche JP, Chiliza B, Emsley R. Insight and

white matter fractional anisotropy in first-episode schizophrenia. Schizophr

Res. 2017 May;183:88-94. doi: 10.1016/j.schres.2016.11.005. Epub 2016 Nov 23.

PMID: 27887780.

23: Kilian S, Burns JK, Seedat S, Asmal L, Chiliza B, Du Plessis S, Olivier MR,

Kidd M, Emsley R. Factors Moderating the Relationship Between Childhood Trauma

and Premorbid Adjustment in First-Episode Schizophrenia. PLoS One. 2017 Jan

20;12(1):e0170178. doi: 10.1371/journal.pone.0170178. PMID: 28107388; PMCID:

PMC5249082.

24: Ovenden ES, Drögemöller BI, van der Merwe L, Chiliza B, Asmal L, Emsley RA,

Warnich L. Fine-mapping of antipsychotic response genome-wide association

studies reveals novel regulatory mechanisms. Pharmacogenomics. 2017

Jan;18(2):105-120. doi: 10.2217/pgs-2016-0108. Epub 2016 Dec 19. PMID: 27992301.

25: Drögemöller BI, Emsley R, Chiliza B, van der Merwe L, Wright GE, Daya M,

Hoal E, Malhotra AK, Lencz T, Robinson DG, Zhang JP, Asmal L, Niehaus DJ,

Warnich L. The identification of novel genetic variants associated with

antipsychotic treatment response outcomes in first-episode schizophrenia

patients. Pharmacogenet Genomics. 2016 May;26(5):235-42. doi:

10.1097/FPC.0000000000000213. PMID: 26928376.

26: Chiliza B, Ojagbemi A, Esan O, Asmal L, Oosthuizen P, Kidd M, Gureje O,

Emsley R. Combining depot antipsychotic with an assertive monitoring programme

for treating first-episode schizophrenia in a resource-constrained setting.

Early Interv Psychiatry. 2016 Feb;10(1):54-62. doi: 10.1111/eip.12141. Epub 2014

Apr 1. PMID: 24690088.

27: Emsley R, Asmal L, du Plessis S, Chiliza B, Kidd M, Carr J, Vink M. Dorsal

striatal volumes in never-treated patients with first-episode schizophrenia

before and during acute treatment. Schizophr Res. 2015 Dec;169(1-3):89-94. doi:

10.1016/j.schres.2015.09.014. Epub 2015 Oct 4. PMID: 26441006.

28: Olivier MR, Killian S, Chiliza B, Asmal L, Schoeman R, Oosthuizen PP, Kidd

M, Emsley R. Cognitive performance during the first year of treatment in first-

episode schizophrenia: a case-control study. Psychol Med. 2015

Oct;45(13):2873-83. doi: 10.1017/S0033291715000860. Epub 2015 May 22. PMID:

25998030.

29: Emsley R, Asmal L, Chiliza B, du Plessis S, Carr J, Kidd M, Malhotra AK,

Vink M, Kahn RS. Changes in brain regions associated with food-intake

regulation, body mass and metabolic profiles during acute antipsychotic

treatment in first-episode schizophrenia. Psychiatry Res. 2015 Aug

30;233(2):186-93. doi: 10.1016/j.pscychresns.2015.06.014. Epub 2015 Jul 2. PMID:

26184461.

30: Chiliza B, Asmal L, Kilian S, Phahladira L, Emsley R. Rate and predictors of

non-response to first-line antipsychotic treatment in first-episode

schizophrenia. Hum Psychopharmacol. 2015 May;30(3):173-82. doi:

10.1002/hup.2469. Epub 2015 Mar 11. PMID: 25758549.

31: Chiliza B, Asmal L, Oosthuizen P, van Niekerk E, Erasmus R, Kidd M, Malhotra

A, Emsley R. Changes in body mass and metabolic profiles in patients with first-

episode schizophrenia treated for 12 months with a first-generation

antipsychotic. Eur Psychiatry. 2015 Feb;30(2):277-83. doi:

10.1016/j.eurpsy.2014.11.013. Epub 2015 Jan 7. PMID: 25577186.

32: Drogemöller BI, Niehaus DJ, Chiliza B, van der Merwe L, Asmal L, Malhotra

AK, Wright GE, Emsley R, Warnich L. Patterns of variation influencing

antipsychotic treatment outcomes in South African first-episode schizophrenia

patients. Pharmacogenomics. 2014 Feb;15(2):189-99. doi: 10.2217/pgs.13.218.

PMID: 24444409.
